# Movement Improves the Quality of Temporal Perception and Decision-Making

**DOI:** 10.1523/ENEURO.0042-19.2019

**Published:** 2019-08-20

**Authors:** Martin Wiener, Weiwei Zhou, Farah Bader, Wilsaan M. Joiner

**Affiliations:** 1 George Mason University, Fairfax, Virginia 22030; 2 University of California, Davis, Davis, California 95616

**Keywords:** decision-making, movement, perception, time perception

## Abstract

A critical aspect of behavior is that mobile organisms must be able to precisely determine where and when to move. A better understanding of the mechanisms underlying precise movement timing and action planning is therefore crucial to understanding how we interact with the world around us. Recent evidence suggests that our experience of time is directly and intrinsically computed within the motor system, consistent with the theory of embodied cognition. To investigate the role of the motor system, we tested human subjects (*n* = 40) on a novel task combining reaching and time estimation. In this task, subjects were required to move a robotic manipulandum to one of two physical locations to categorize a concurrently timed suprasecond. Critically, subjects were divided into two groups: one in which movement during the interval was unrestricted and one in which they were restricted from moving until the stimulus interval had elapsed. Our results revealed a higher degree of precision for subjects in the free-moving group. A further experiment (*n* = 14) verified that these findings were not due to proximity to the target, counting strategies, bias, or movement length. A final experiment (*n* = 10) replicated these findings using a within-subjects design, performing a time reproduction task, in which movement during encoding of the interval led to more precise performance. Our findings suggest that time estimation may be instantiated within the motor system as an ongoing readout of timing judgment and confidence.

## Significance Statement

Perception is inherently a noisy process, wherein the nervous system must find a tradeoff between measurement noise, bias, distractions, and expectations. Recent evidence has begun to demonstrate that our perceptions and sensations are influenced by motor movements and actions. One area where this is of particular importance is the perception of time. Recent work has shown that our temporal perception fluidly changes with the length of our movements. Further, there is also evidence suggesting that movement not only biases perceived time, but can enhance it, suggesting the motor system directly influences temporal perception. We here show that when subjects are free to move during the timing of an auditory interval, the representation of that interval is enhanced.

## Introduction

A critical aspect of behavior is that mobile organisms must be able to precisely determine where and when to move (Rosenbaum, 2009). A better understanding of the mechanisms underlying precise movement timing and action planning is therefore crucial to understanding how we interact with the world around us. A majority of studies focusing on action planning and reaching movements have been focused over the past 30 years on spatial aspects of reaching (Wolpert et al., 2011). Specifically, there is interest regarding the locations where subjects will move to, and how subjects will update their movements to particular locations when the targets they are reaching for are either unknown or changed. These studies further focus on motor adaptation paradigms, in which the parameters of a movement trajectory are altered, unknown to the subject, such that a subject does not arrive at the location they expected to, and so must update their internal model of movement to accommodate these changes (Sing et al., 2009; Wei and Körding, 2009; Gallivan et al., 2017; Hosseini et al., 2017). The results of these studies provide important insights into how the motor system both adapts and accommodates changes in movement trajectories, and have provided dissociations between the preparation, planning, and executions of movements (Shadmehr et al., 2010; Haith et al., 2016; Wong et al., 2016).

A critical aspect of the previous studies is the timing of motor movements. Indeed, timing is inexorably tied to movement, as for any movement to take place, temporal precision is needed to coordinate between different limb movements and the environment (Todorov, 2004). Remarkably, recent studies show that our perception of time depends on whether we are moving or not (Hagura et al., 2012; Tomassini et al., 2012, 2014; Press et al., 2014; Yokosaka et al., 2015; Tomassini and Morrone, 2016; Imaizumi and Asai, 2017; Yon et al., 2017). Prolonged movements serve to expand the perceived duration of a concurrently presented stimulus (Yokosaka et al., 2015), and perceptual judgments of time tend to gravitate toward the length of our motor movements (Press et al., 2014), even when these movements are not tied to the task in any way (Yon et al., 2017). These distortions can also occur when viewing the movements of other people, and can further be altered by the sense of agency a viewer has during the observation (Imaizumi and Asai, 2017). Further, even the preparation of a movement is sufficient to induce temporal distortions, such that stimuli are perceived as longer when we are preparing to move than when not (Hagura et al., 2012). These distortions are also dependent on both the direction and speed of movement being planned or executed (Yokosaka et al., 2015; Tomassini and Morrone 2016). Finally, movement distortions affect concurrently perceived stimulus durations regardless of the stimulus modality in which they are presented (Tomassini et al., 2012, 2014; Tomassini and Morrone 2016), suggesting that motor movements serve a supramodal role in the processing of temporal duration.

Although the previous findings suggest that movements can serve to distort perceptual timing, there is also evidence to suggest that it can enhance it. Indeed, evidence has shown that, during movement preparation, in addition to an expanded perception of time, there is also an increase in temporal fidelity, such that subjects can more easily perceive visual flicker and rapidly presented stimuli (Hagura et al., 2012). Similarly, when time perception for a particular modality has been altered, such as via adaptation, concurrent movements can serve to reorient subjects to the appropriate duration (Tomassini et al., 2012). These findings suggest that, when concurrent movement is engaged or simulated, the timing of environmental stimuli adopts computations of the motor system. However, whether this adoption is because of timing being overwritten by the motor system, or enhanced by it remains unknown.

To determine the involvement of the motor system in temporal perception and decision-making, we tested human participants on four tasks combining time perception and reaching movements. Our findings indicate that movement during concurrent timing can enhance temporal representations.

## Materials and Methods

### Participants

A total of 64 healthy subjects (30 male and 34 female, 23.5 ± 0.56 years of age) with no known neurologic disorders or impairments were recruited for this study. All participants were right-hand dominant and used this hand to perform the task throughout the entirety of the experiment. Each participant only performed a single experimental paradigm and was naïve to the task. All study protocols were reviewed and approved by the institutional review board according to standard regulations and guidelines; all participants signed an informed consent before performing any task.

### Apparatus

**Figure 1.**
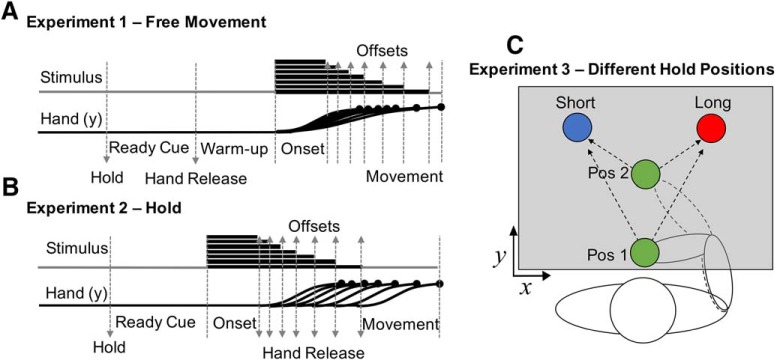
Task design for the three experimental types. In each experiment subjects held the handle of a robotic manipulandum and moved a screen cursor to one of two targets to classify a tone as short (left target) or long (right target). ***A***, In Experiment 1 (free-movement), subjects were held in place in the starting position, while a ready cue was presented. Following this, the arm was released for a warm-up period, in which subjects could freely move. Onset of the tone proceeded for one of seven possible durations (log-spaced), and subjects were free to move to one of two target locations, but could only enter a location after tone offset occurred. ***B***, In Experiment 2 (hold) no warm-up period was provided, and subjects were only released once the tone duration was complete. ***C***, In Experiment 3 (different hold positions) subjects performed the hold experiment, but with one of two possible starting positions, in different blocks.

Participants were seated in an adjustable chair directly in front of the robotic manipulandum (KINARM End-Point Lab, BKIN Technologies) at a height where their forehead could rest comfortably on the system’s headrest. A horizontal mirror display occluded the subject’s view of the right forearm to limit feedback of the upper limb and hand position to only what was observed on the screen. Visual feedback from the task was projected onto the mirror from a downward-facing LCD monitor positioned directly above. Participants gripped the right handle of the robotic manipulandum and made reaching movements to one of two circular targets 0.5 cm in diameter, placed 14 cm apart on the sagittal axis of the body (left target is the short duration location and right target is the long duration location; Fig. 1*C*). During movements, the manipulandum continuously measured hand position, velocity, and forces applied by subjects while simultaneously exerting external forces at a sampling rate of 1000 Hz.

### Experiment 1: free-movement

In the first experiment (Fig. 1*A*), termed the “free-movement” group, subjects (*n* = 20) were first instructed on each trial to move the robotic manipulandum so that the screen cursor was brought to a starting location, located 10 cm away from each target. Once there, the manipulandum was locked into place and subjects were unable to move the handle. Subjects remained locked in this position for 1000 ms while the words “Get Ready” were presented 10 cm under the starting location. Next, the short and long duration locations were presented to the subject; both cues were equidistant from the starting position, located at 105° and 75°, respectively. The choice of a left/right orientation for short/long responses was chosen to avoid any incongruence effects that may arise from spatial associations; previous studies have shown that left-sided stimuli are more typically associated with “shorter” temporal durations, adhering to a left-right mental timeline; the so-called STEARC (spatial-temporal association of response codes) effect (Ishihara et al., 2008). After, the ready cue disappeared, the starting location turned blue and the robotic manipulandum was released from the hold position, allowing subjects to freely move. This “warm-up” period lasted for 2000 ms, after which a tone was played via speakers to the subject. Tone durations were logarithmically spaced between 1 and 4 s in seven steps, and presented in a first-order counterbalanced sequence (Aguirre et al., 2011) for a total of 56 trials for each duration (392 trials total divided into 8 blocks of 49 trials each). Once the tone duration had elapsed, subjects were required to classify it into short or long duration categories. Subjects were instructed to judge each duration relative to the average of all previously presented durations. Notably, if a subject moved into either the short or long duration target locations during the warm-up or any other time before the to-be-presented duration had elapsed, the two duration locations disappeared and subjects were required to start the trial over again. All subjects were additionally instructed that they must respond as quickly, yet as accurately, as possible. Once subjects made a choice, the corresponding target turned green and then all stimuli were removed. No feedback was provided. A new trial was initiated after a 500 ms intertrial interval. Given the speeded nature of the task, an ideal strategy would be to start by moving the cursor as close as possible to the “short” target, and then move the cursor over toward the “long” target as the interval elapsed.

### Experiment 2: hold

In the second experiment (Fig. 1*B*), termed the “hold” group, a different set of subjects (*n* = 20) performed the same task as the free-movement group, except that no warm-up period was provided, and the cursor was not released from the hold position until the offset of the tone duration. Thus, unlike the warm-up period in the free-movement group, subjects were not allowed to move into a readied position to respond (i.e., enter the short or long duration location).

### Experiment 3: different hold locations

In the third experiment (Fig. 1*C*), termed the “different hold locations” group, a different set of subjects (*n* = 14) performed the same task as the hold group, except that start position changed across blocks (close start: 7.8 cm away from the duration category targets; far start: 10 cm away from the targets). As in the hold experiment, the cursor was not released from the hold position until the offset of the tone duration. In this condition, subjects completed twice as many trials as in the previous experiments, with 392 trials for each of the starting locations (784 trials total).

### Experiment 4: temporal reproduction

In the fourth, temporal reproduction experiment, each trial contained two phases. In the first (encoding phase) subjects were presented with a tone of random duration (1000, 1500, 2000, 2500, 3000, 3500, and 4000 ms). In the second (reproduction phase) subjects were required to reproduce the tone duration with their hand movement. At the beginning of each trial, subjects (*n* = 10) were first instructed to move the robotic manipulandum to a presented start location (represented by a white circle) which was randomly selected from 9 possible locations (locations were on a 3 × 3 grid square, 10 cm in length). Once there, the manipulandum was locked into place and subjects were unable to move the handle. Subjects remained locked in this position while the words “Get Ready” were presented 10 cm under the starting location. After a 1000 ms delay, the ready cue disappeared. This represented the beginning of the “encoding phase” and a tone (440 Hz) was played for a random duration selected from the seven durations listed above. There were two conditions for the encoding phase: (1) subjects were allowed to freely move while listening to the tone, termed the “encoding free-movement” condition; (2) subjects were locked at the start location when the tone was played, termed the “encoding hold” condition. On encoding free-movement trials, the manipulandum was released at the onset of the tone and the start location turned green to cue the subjects to move. At the offset of the tone, subjects were asked to stop moving and the robot returned the handle back to a new start location which was randomly selected from the remaining eight possible start locations (i.e., the first start location was not repeated). On encoding hold trials the handle was locked at the start location for the entire tone duration and subjects did not move the manipulandum. In both conditions the handle was locked after the tone duration for a short interphase interval (randomly selected between 450 and 550 ms). Following the interphase interval subjects entered the “reproduction phase” of the trial and were instructed to move the handle when the start location turned green and stop when they felt they had moved for the duration of the tone played during the encoding phase. All stimuli, including the feedback cursor, were removed from the screen when subjects made movements, and there were no restrictions on the movement patterns made. Subjects completed a 20 trial familiarization block containing 10 trials with the encoding free-movement condition and 10 trials with the encoding hold condition. This was followed by eight testing blocks of 42 trials each (4 blocks with encoding free-movement and 4 blocks with encoding hold. Each duration was tested 6 times in each block). The order of the testing blocks was counterbalanced across subjects. No feedback was provided.

### Analysis

#### Behavioral data

Behavioral data were analyzed similarly to earlier work with this paradigm (Wiener et al., 2014). Choice and reaction time data were first calculated for each subject for each of the seven tested durations in our stimulus set; reaction time data were estimated as the difference between the offset of the presented interval and arrival at the target location. Psychometric and chronometric curves were then generated for each participant. Psychometric curves were generated by plotting the proportion of long response choices for each of the seven tested durations; these points were then fitted by a cumulative Gumbel distribution using the *psignifit* v3.0 software package (http://psignifit.sourceforge.net/) for Python (Fründ et al., 2011). The Gumbel distribution was chosen to reflect the log-spacing of the duration stimuli, as well as the uncertainty associated with longer stimulus durations (van Driel et al., 2014). Upper and lower thresholds, the approximate points at which the subject is 25 or 75% likely to judge the stimulus as long, were calculated using the bias-corrected bootstrap method implemented by *psignifit*, based on 1999 simulations. The results of this analysis yielded the bisection point (BP; the time value at which subjects were equally likely to judge the stimulus as long or short), the difference limen [DL; the difference between the upper (75%) and lower (25%) threshold values divided in half], and the coefficient of variation (CV; DL/BP). The BP thus reflects the subjective midpoint of the range of tested durations, whereas the CV reflects the normalized variability of measurements. Chronometric curves were constructed by plotting the reaction time for each of the seven possible durations.

#### Movement data

Movement data were extracted for all experimental conditions by calculating the *X* and *Y* positions of the manipulandum, as well as the force applied in each direction. Force direction data were calculated by the inverse tangent of *X* and *Y* force data using MATLAB’s *atan2d* function. To calculate a reference point for the free-movement condition, we measured the Euclidean distance between the *X*–*Y* coordinate and the location of the short and long duration targets, and then took the ratio of the two (short/long). For movement length, we calculated the average Euclidean path length of movement during each of the seven intervals in our stimulus set.

As a measure of variability for both movement conditions, we calculated the average intertrial SD of movement location. This was done by first selecting data only for the last 100 ms of each trial, and then calculating the SD of the *X* and *Y* positions across trials for every 1 ms time point leading up to arrival at the target location. We then calculated the average SD across this 100 ms interval for each of the seven stimulus intervals in our stimulus set.

To calculate changes-of-mind in our dataset, we began by calculating two vectors from the movement initiation location. The first (*V1*) was calculated as the vector from the movement initiation (when hand velocity exceeded 5 cm/s) to the location of max velocity. The second (*V2*) was calculated as the vector from the point of max velocity to the target location that subjects chose on that trial. These vectors were then used to calculate angle θ as the angle between *V1* and *V2*. If a subject continues from the start location to the same target, then V1 and V2 will proceed to the same point and θ will be small, but will be large if the subject changes to a different target. We settled on a criterion of θ > 40°, based on preliminary analysis and visual confirmation that this criterion accurately identified change-of-mind trials. We note that this method is similar to that used by Resulaj et al. (2009) to identify change-of-mind trajectories.

For temporal reproduction data, estimated durations were calculated as the difference in time between when subjects started moving and stopped moving during the reproduction phase. Average reproduced durations were calculated for each tested interval. To measure variability, we calculated the CV as the SD of reproduced durations divided by the mean reproduced duration; CVs were calculated separately for each tested interval. To measure path similarity, we calculated the discrete Fréchet distance. The exact Fréchet distance is a NP-hard problem, and so cannot reliably be calculated in polynomial time; however, the discrete Fréchet distance algorithm allows for an approximation of this value between both trajectories. We calculated this value between encoding and reproduction phases across all trials for each subject using the *DiscreteFrechetDist* function for MATLAB, available at (https://www.mathworks.com/matlabcentral/fileexchange/31922-discrete-frechet-distance), which implements the algorithm by (Eiter and Manilla, 1994).

### Statistical analyses

For all bivariate comparisons between groups, we conducted two-tailed Mann–Whitney *U* tests. For within-subject bivariate comparisons, two-tailed Wilcoxon signed rank tests. In both cases, alpha was set at 0.05, and all *p* values were confirmed with 10,000 permutations to generate 95% confidence intervals. For comparisons of reaction time, where duration was a factor, mixed-model ANOVAs were run with duration as a within-subject factor and group as a between-subject factor. For Experiment 3 (different hold locations), duration and distance were the only within-subject factors. Measures of effect size were calculated for all significant effects.

### Logistic regression analysis

To conduct regression for choice data based on single-trial movement trajectories and force data, several steps were taken. First, a softmax transform was applied to the ratio of short/long distance values for the free-movement group, and the force direction data for the hold group, to constrain extreme values and bring both sets into a comparable reference frame. Next, we extracted these data in 1 ms steps for the 1000 ms before offset for each of the seven intervals in our stimulus set. This timeframe was chosen as it represents the minimum number of time points that can be viewed across all seven intervals, as the shortest interval in our stimulus set was 1000 ms. Then, for each 1 ms time point, we constructed a generalized linear mixed-effects model, using the *fitglme* function for MATLAB. Initially, each model was constructed such that the choice on any trial (Y) could be predicted by a linear combination of the duration presented on that trial (D) and the normalized short/long ratio for free-movement and the normalized force direction for the hold group (M) as fixed effects, with individual subject (S) as a random effect, resulting in the function [Y ∼ 1 + D + M + (1S)] in Wilkinson notation. A binomial distribution was chosen to model the response distribution of choices, with a logit curve as the link function. Fitting of each model was accomplished using the Laplace approximation for Maximum Likelihood, as optimized by the MATLAB function *fminsearch.* The result of this analysis yielded 1000 tests for each time point for free-movement and hold groups. To account for multiple comparisons, we applied a Bonferroni correction to maintain a test-wise alpha level of 0.05, leading to an adjusted alpha of 0.00005. Following our initial analysis, we repeated our logistic regression analysis using only data from the 2000 ms interval trials. In this case, the duration of the trial is always fixed, and so we calculated a reduced function without duration, using only the movement parameter for that subject. We again repeated this analysis using 1000 ms. An additional analysis was run on the pre-onset time period for the hold group, which spanned 2000 ms.

## Results

**Figure 2.**
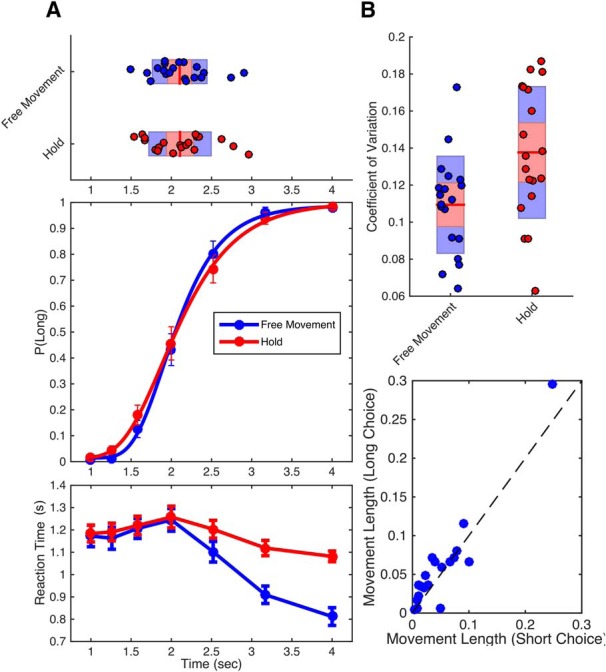
Behavioral data from free-movement and hold experiments. ***A***, Middle, The average proportion of trials on which subjects classified durations as long as a function of tested duration. Psychometric curves represent fits to the average data. Red symbols represent data from Experiment 2 (hold); blue symbols represent data from Experiment 1 (free-movement). Bottom, Average chronometric data, with reaction time as a function of duration. Reaction time was notably similar for both groups for durations under 2 s, but then became increasingly faster for free-movement subjects. Top, Individual bisection points, derived from fitted curves. Boxplots display the mean (red line), bounded by the 95% confidence interval (red shaded region) and the SD (blue region). ***B***, Top, CV data for both groups, demonstrating significantly lower variability for the free-movement experiment than the hold experiment. Bottom, The average movement length for subjects in the free-movement experiment for 2 s trials classified as long or short; subjects chose long more often when they moved more during the 2 s tone interval. Dashed line represents the identity. Results remained significant with removal of the outlier. Error bars represent SE.

Our analysis began by measuring psychometric and chronometric functions, in which we measured the proportion of trials in which subjects classified each duration as long, and the reaction time for each duration, respectively. Psychometric data were additionally fit with a cumulative gumbel distribution, using the bias-corrected bootstrap method outlined by Wichmann and Hill (2001) and implemented in the *psignifit* toolbox (Fründ et al., 2011). From this curve, the BP was determined as the duration corresponding to a 0.5 probability of classifying the duration as long. Additionally, the CV was determined as the difference between the 0.75 and 0.25 probability durations (difference limen) divided by the BP. Our initial analysis of these data revealed that subjects in the free-movement group were significantly less variable than subjects in the hold group (Fig. 2), as characterized by a lower CV (Mann–Whitney *U* = 124, *p* = 0.012, Cohen’s *d* = 0.69, 10,000 permutations), with no change in the BP (Mann–Whitney *U* = 196.5, *p* = 0.925; see Fig. 4). We further confirmed a similar reduction in the difference limen values (Mann–Whitney *U* = 120, *p* = 0.046), suggesting that this difference was not an artifact of the CV calculation. Additionally, RTs were significantly lower in the free-movement group than in the hold group (duration by group interaction: *F*_(6228)_ = 15.743, p < 0.001, η^2^_p_= 0.293), which had been expected, given that these subjects were allowed to move closer to the targets during tone presentation; yet, unexpectedly, the RT advantage only held for durations longer than the BP (>2000 ms), with comparable RTs for durations classified as short. These findings suggest that the allowance of movement during timing conveys a perceptual advantage in temporal bisection.

**Figure 3.**
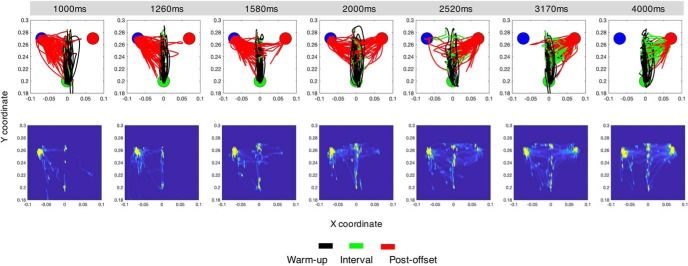
Movement trajectories for an example subject in Experiment 1 (free-movement). Top row, Layout of the start position (green filled circle), and short (blue filled circle) and long targets (red filled circle) is the same as in Figure 1*C*. Distance is in meters. Each panel represents the total number of movements for one of seven intervals used in our temporal bisection task. Color-coding of traces reflects hand movement through the progression of the given trial (black, warm-up period; green, during the tone duration; red, after the tone until the decision). As can be observed, for relatively short durations (<2000 ms), the majority of movements smoothly progress from the starting position at the bottom of the panel to the short target location (left target). As durations increase (1580, 2000, and 2520 ms), choices split between the short and long locations (note the directions of the red traces). Notably, for relatively long durations (>2000 ms), subject arm movements initially move toward the short location, but then shift toward the long location (right target) once the interval has elapsed past a certain point. Bottom row, Density estimates for arm location only during the interval portion of each trial (brighter pixels represent a greater proportion of the trial occupied in that location), displaying the shift to the long duration target with longer intervals.

**Figure 4.**
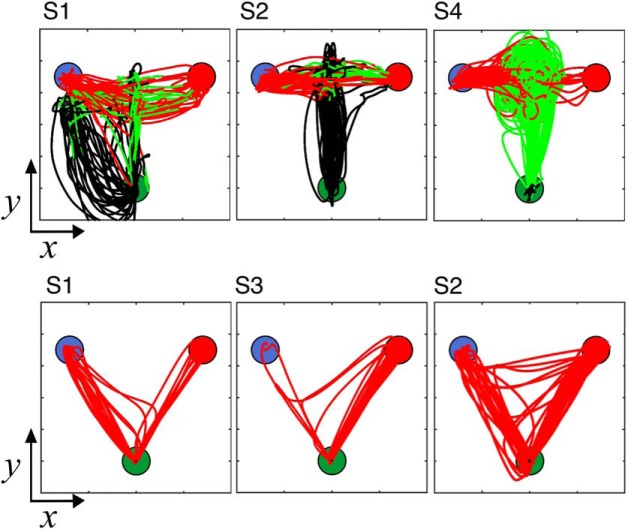
Movement trajectories are idiosyncratic between subjects, yet consistent within subject. Layout of the start position (green filled circle), and short (blue filled circle) and long targets (red filled circle) is the same as in Figure 1*C*. Color-coding of traces reflects hand movement through the progression of the given trial (black, warm-up period; green, during the tone duration; red, after the tone until the decision). Top row, Trajectory data for 2000 ms trials for three example subjects from Experiment 1 (free-movement). Classification of a short duration is the left target; a long classification is the right target. Each subject employed a separate strategy, with one subject (S4) moving in a circular pattern between short and long locations, another (S1) rotating in a leftward arc before moving in between both targets, and a third (S2) moving in an up–down fashion before shifting from the middle to a target location. Bottom row, Three example subjects for 2000 ms trials from Experiment 2 (hold). Changes-of-mind are evident, including a shift from long to short (S1), short to long (S3), and both (S2).

Why might subjects perform better during free-movement than the hold condition? Examination of the movement trajectories revealed a complex picture into the deliberation process. As expected for the ideal strategy, some subjects moved the cursor closer to the short duration target initially, but then gradually moved closer to the long duration target as the interval on a given trial elapsed (Fig. 3). However, across subjects, examination of movement trajectories revealed that nearly every subject adopted a slightly different strategy (Fig. 4). Some subjects adopted a “circular” strategy, where they spun the arm in a circle during the warmup and duration presentation phases (Fig. 4, S4); others chose a “middle” strategy, keeping the cursor in between the short and long locations and hedging closer to one location or the other (Fig. 4, S1). Still others chose an “up–down” strategy, where they moved the cursor along the midline up and down between the two targets before moving to a target at offset (Fig. 4, S2). Thus, subject trajectories were idiosyncratic, yet consistent within each subject. Given the patterned nature of performance, where some free-movement subjects moved the cursor in a rhythmic-like fashion, one possibility is that subjects were using the arm to adopt a counting strategy. As counting can lead to reductions in the CV (Hinton and Rao, 2004), this may have accounted for the improvement in performance. We analyzed this possibility by collecting the average path length traveled for subjects across trials during each duration. As such, subjects who moved in a rhythmic fashion would have a longer path length than subjects who kept the cursor in a relatively stable location or chose the ideal strategy. These subjects should also have lower CVs than subjects with no patterned movement. Although a negative relationship was observed, no significant correlation between mean path length and CV was found (Spearman rho = −0.184, *p* = 0.446, 10 000 permutations), suggesting that the improvement in CV was not because of a counting/rhythmic strategy. Additionally, no between-subject correlation was found between average movement length and the BP (rho = 0.279), indicating that movement length did not overall shift bias in responses; however, we note that these findings do not entirely rule out sub-vocal counting as an explanation for the effect on this task.

Although no effect between subjects was found on the CV, there is the possibility that movement length did still influence choice within subject. Here, we extracted mean movement length for trials classified as short versus long, only for the middle duration of the stimulus set (2000 ms), at the approximate location of the BP and the most ambiguous point. We observed an impact of movement length on 2000 ms trials, with longer movement lengths during the interval when subjects chose long than when they chose short (Wilcoxon signed rank test, *Z* = −2.173, *p* = 0.027, Cohen’s *d =* 0.753, 10,000 permutations; Fig. 2*B*, bottom), consistent with previous reports that longer movements are associated with longer perceived intervals (Yokosaka et al., 2015).

### Changes of mind

For the hold group, although movement trajectories were constrained to ballistic movements to the target at interval offset, notable intersubject differences were observed (Fig. 4, bottom row). The most salient feature here was that these subjects exhibited the same “change-of-mind” pattern as observed in other recent reach-to-touch decision-making tasks (Resulaj et al., 2009; van den Berg et al., 2016). To investigate this further, we quantified changes of mind on a trial-by-trial basis (see Materials and Methods). We observed that, although the majority of trials were characterized by direct movements to a target, an observable subset of trials contained trajectories that began toward one target, and then switched in direction to a different target (Mean proportion = 0.034 ± 0.007 SE). These changes-of-mind occurred in both directions (short-to-long; long-to-short), and notably occurred more often for some subjects than others (see Fig. 6). Previous research has suggested that these changes occur as a re-evaluation of the given evidence after a stimulus has been presented, and further show that, when a subject changes their mind, they often change it to the correct answer (Resulaj et al., 2009). In the temporal bisection task, a “correct” choice is relative to the criterion used for classification, which may vary by subject.

**Figure 5.**
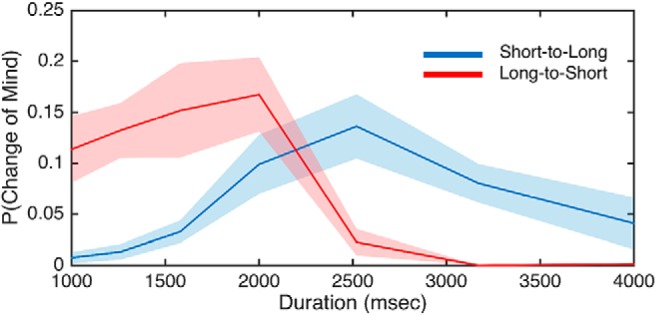
Changes of mind occur in the appropriate direction. Displayed are the mean proportion of changes-of-mind that occurred for each interval in our stimulus set for the hold group for each transition direction. Accordingly, when subjects switched from the long to the short target, it was generally for intervals <2000 ms, whereas the opposite pattern presented for changes from the short to the long target location. No significant difference was observed for the middle interval (2000 ms). Shaded areas represent SE.

One possible explanation for the difference in precision between free-movement and hold subjects is that the movement restriction of the hold task induces more changes-of-mind, by delaying movement until after the interval has elapsed (Haith et al., 2016). Although the proportion of changes was low, we addressed this possibility by removing all change-of-mind trials and comparing bisection performance. We once again detected a significant difference in the CV between groups (Mann–Whitney *U* = 112, *p* = 0.027, Cohen’s *d* = 0.75), indicating that changes-of-mind could not explain the difference in precision. Further, we quantified the proportion of changes-of-mind in either direction (short-to-long; long-to-short) for each interval in our stimulus set. A repeated-measures ANOVA revealed a significant interaction between the direction of the shift and duration (*F*_(6114)_ = 9.589, *p* < 0.001, η2p = 0.335; notably, changes-of-mind, when they occurred, generally occurred in the appropriate direction; Fig. 5). That is, short-to-long transitions occurred for intervals greater than the midpoint (2000 ms) whereas long-to-short transitions occurred for intervals less than the midpoint. Additionally, no significant difference in transition direction was observed for the middle interval itself (Wilcoxon signed rank test, *Z* = −1.445, *p* = 0.157).

### Movement dynamics inform temporal estimates

**Figure 6.**
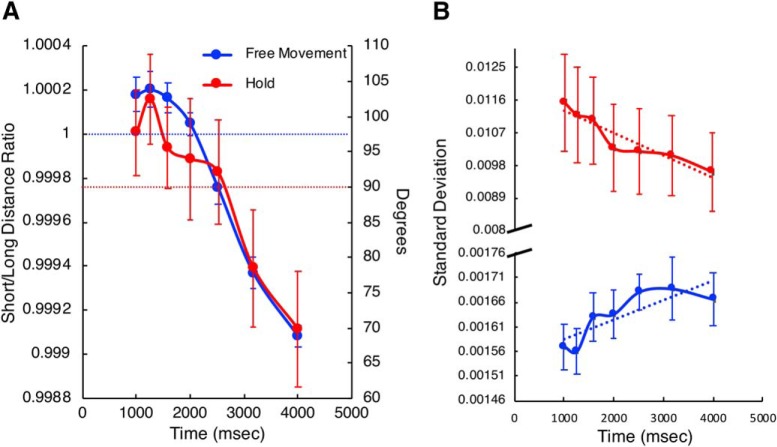
The decision process is represented within the movement trajectories and force patterns. ***A***, Position and force data for both experiments as a function of tone duration. Red symbols represent data from Experiment 2 (hold); blue symbols represent data from Experiment 1 (free-movement). The left axis displays the ratio of the Euclidean distance from arm position to the long and short targets for the free-movement group; values >1 (horizontal blue dashed line) indicate the subject was closer to the short location, and vice versa. The right axis displays the force direction, in degrees, that subjects were exerting on the arm at tone offset. Values above 90° (horizontal red dashed line) indicate the subject was pushing toward the short target (105°), whereas below 90° indicate pushing toward the long target (75°). ***B***, Intertrial variability (SD) of the arm position was measured for the final 100 ms of arm movements across trials for each duration. Free-movement subjects (blue trace) demonstrated linearly increasing SD with tone duration, whereas hold subjects (red trace) displayed linearly decreasing SD. Note the axis break and scale difference in the *y*-axis, demonstrating that hold subjects exhibited greater variability overall compared with free-movement subjects. Error bars represent SE.

**Figure 7.**
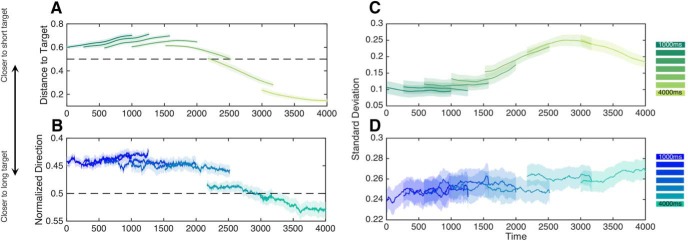
Differences in movement dynamics across tasks. Movement dynamics for the free-movement group (***A***) and hold group (***B***) during the presentation of each interval. Data are shown for the last 1000 ms of each presented interval. Dashed lines represent the 50% inflection point between the long and short duration targets, with values above this line representing a closer relative location (top) or a closer force direction (bottom) to the short target location. All displayed data are sigmoid transformed to reduce the influence of extreme values on logistic regression (see Materials and Methods). For both sets, movement dynamics display a transition from proximity to the short duration target to the long duration target as the interval elapses; additionally, differences in position or force could be distinguished well before the interval offset. ***C***, Intertrial variability of movement dynamics displayed in free-movement and the hold group (***D***). Both panels display the average within-subject SD of movement data from their corresponding left panels. For both groups, intertrial variability increases as the presented interval elapses, but with different profiles. Further, variability is dramatically lower for the free-movement group compared with the hold group (note the difference in the vertical scale). Shaded regions display SE.

In addition to the above findings, our results suggested that the subject’s choice could be determined by the movement dynamics (see Fig. 7). To determine this, we first examined the location of the cursor at the offset of the duration signal in Experiment 1 (free-movement). For each trial, we calculated the Euclidian distance of the cursor to the short and long targets, and then took their ratio (short distance/long distance); values >1 indicate the cursor was closer to the short target. Here, we found that, regardless of strategy used, free-movement subjects were closer to the short-duration target at the shortest interval offset, and then gradually shifted toward the long-duration target with longer intervals (*F*_(6114)_ = 58.912, *p* < 0.001, η^2^_p_ = 0.756). As expected, this finding suggests that subjects decided which choice they will make before the target duration had elapsed. For the hold group, the position at offset would provide no information, as subjects were held in place until this point in time. Instead, we measured the direction of force being applied to the robotic manipulandum arm handle by the subject; if the force direction is informative of choice, then this should be interpretable by the orientation of force toward one of the two targets. We further note that force information would be less informative in the free-movement condition, as subjects may apply directional force in proximity to one of the targets while not directly facing it. On each trial, we calculated the force being applied in *x* and *y* coordinates, and the resultant direction subjects were pushing in. Here, we found that subjects were pushing in the direction of the short target location (105°) for the shortest duration, and then gradually shifted their direction toward the long target location (75°) with increasing duration (*F*_(6114)_ = 10.720, *p* < 0.001, η^2^_p_=0.361). Both effects were linear, and showed a remarkable degree of correspondence between experimental groups (Fig. 6*A*). A noteworthy feature of this finding is that it indicates that the choice was not simply binary; that is, subjects did not adjust force in a stepwise fashion, as would be expected if motor output simply reflected the crossing of an internal categorical boundary. In other words, motor output did not merely represent the endpoint of the decision. Instead, subjects moved along a gradient between the two target locations. This gradient provides a readout of elapsed time from the subject’s movement, and also further highlights the uncertainty subjects had in classifying temporal durations, as durations near the middle of the stimulus set were also near the respective middle locations. Further, it suggests the motor system is continuously evaluating the length of an interval, and can be used as a measurement of subjective internal timekeeping.

Consistent with the above explanation, we examined the variability of movement trajectories as subjects were approaching their chosen target. We measured the intertrial variability of movement trajectories in the last 100 ms of each trial’s movement (Fig. 6*B*). Here, we again found a remarkable difference between groups. For the free-movement group, the average movement variability increased linearly with increasing durations (*F*_(6114)_ = 2.287, *p* = 0.04, η^2^_p_=0.207), such that subjects were more variable in their movements when committing to a longer duration choice. Yet, for the hold group, we found the exact opposite pattern; subjects became less variable in their movements with longer durations (*F*_(6114)_ = 4.638, *p* < 0.001, η^2^_p_=0.196). Additionally, these subjects were dramatically more variable overall in their movement trajectories than in the free-movement group, despite the fact that these measurements were collected from the last 100 ms of movement in both groups. One reason for this difference may have been the distance subjects must travel when making a choice. Indeed, movement trajectories are commonly more variable in reaching for further targets (Messier and Kalaska, 1999). Yet, the different relationships between variability with increasing duration cannot alone be explained by this. As subjects were also faster at making reaching movements after longer durations, we suggest a distance/speed tradeoff in variability is the source of this effect. That is, if a target is far away, progressively faster (and more certain) movements become less variable, whereas if a target is close, faster movements are more variable. This finding follows kinematic theories of movement (Plamondon and Alimi, 1997), which suggest a nonlinear effect of the speed/accuracy tradeoff for different movement distances (Hancock and Newell, 1985).

To further examine the effects of force and distance ratios in our two experimental groups, we performed a logistic regression analysis of single trial responses with the movement parameters described above. Specifically, we constructed a generalized linear mixed effects model in which the response choice (short or long) could be predicted by the long/short distance ratio for the free-movement group, or the force direction data for the hold group. We further repeated this analysis in 1 ms steps for the 1000 ms preceding the offset of the interval for both groups. This time value (1000 ms) reflects the largest amount of time from interval offset that distance/force data were available for all intervals in our stimulus set. The first finding of this analysis was that choices could be accurately decoded for the free-movement group for up to 810 ms before the offset of the interval (all *p* < 0.05, Bonferroni-corrected). This finding extended our analysis of the offset points alone, with the movement trajectories demonstrating a good separation between distance ratios well before offset of the interval (Fig. 7*A*). For the hold group, force data were notably noisier (Fig. 7*B*); significant periods where force direction predicted choice were detected, but not for any consistent period of time, and only at an uncorrected level (*p* < 0.05).

The data further suggests that the momentary position or force exertion throughout the interval can serve as an index of the internal timekeeping process. To that end, we also examined intertrial variability in both measures. If movement dynamics provide a measure of temporal estimation, then the variability of those measurements should index the variability, and uncertainty of timing. More specifically, as the uncertainty of the length of a time interval grows with its duration, the variability of movements during the interval should also grow with duration; if a salient difference between the Hold and free-movement conditions is an improvement in the fidelity of the time estimation process, then we would also expect movement variability to be smaller in the free-movement group during time estimation. Consistently, when examining the SD of either the normalized position between short and long ratios, or the normalized force direction data, we observed a striking increase in variability for both groups that continuously increased throughout the interval. Additionally, two notable differences between free-movement and hold groups were readily observed. First, whereas variability increased linearly in the hold group (Fig. 7*D*), in the free-movement group this increase was slow to start, then rapidly increased before peaking at ∼2800 ms and then decreasing until the end of the 4000 ms interval (Fig. 7*C*). Both increases are consistent with the increasing uncertainty in timing longer intervals; however, the increase in uncertainty for the free-movement group shows a striking violation of linearity, not in accordance with the scalar property of timing. This difference may further explain the difference in CV between groups; the allowance of free-movement improves temporal perception, and may use a different metric for achieving precision outside of scalar timing bounds.

**Figure 8.**
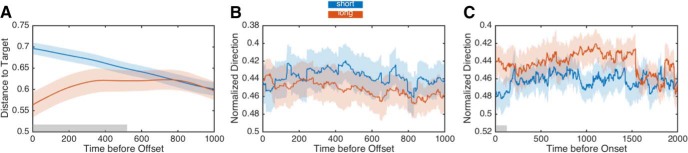
Movement dynamics predict eventual choice. All three panels display data for the middle interval of the stimulus set (2000 ms). ***A***, Normalized movement ratios for the free-movement group. Eventual choice could be determined ∼500 ms before interval offset. ***B***, Normalized force direction for the hold group. In contrast to the free-movement group, no distinguishable difference in direction was observed between short and long response choices. ***C***, Normalized force direction for the hold group during the interval before stimulus onset. Here, a distinguishable difference was detected in the final 100 ms before target onset; note that the direction subjects are exerting force is pointing in the opposite direction of the choice they will eventually make on that trial. Shaded error represents SE. Gray shaded regions indicate significance at *p* < 0.05 for logistic regression.

To further examine the predictability of movement dynamics for indicating timing and decision-making, we again conducted a logistic regression analysis, but this time only for the 2000 ms interval, which was located at the middle of the stimulus set and is thus the most ambiguous (Fig. 8). Accordingly, movement dynamics should indicate which choice the subject will make. For the free-movement group, distance ratios successfully determined which choice the subject would make up to 345 ms before interval offset (all *p* < 0.05, Bonferroni-corrected; 540 ms uncorrected), demonstrating that by 1655 ms or 82% of the way through the interval, the choice the subject would eventually make could be determined. For the hold group, force data revealed a different pattern; we were unable to decode subject choice at any point before offset of the interval, even at the point of offset itself. This finding suggests that, in the hold group, force data provides a less reliable index of the unfolding time estimation and decision-making process. On the other hand, it alternatively suggests that subjects in the hold group were less certain of their decision while timing, matching the decreased precision of interval estimates in this group.

One additional reason for the lack of decoding accuracy in the hold group comes from the presence of change-of-mind trajectories in this group. If subjects changed their mind after the arm is released at interval offset, then the force direction data before offset will only be weakly informative to where subjects will eventually move to. Yet, these changes-of-mind may also be detectable in the force response profiles in the form of bias. To investigate this further, we conducted our logistic regression analysis on force and movement profiles during the 2000 ms period before interval onset of the middle stimulus interval. Here, we found no significant effects in the free-movement group; however, we did detect significant differences in the hold group, specifically during the last 100 ms before stimulus onset (all *p* < 0.05, uncorrected). Examination of the force profiles during this time revealed a separation in the force direction between trials on which subjects would eventually choose short or long. Crucially, we observed that the force direction in this case was in the opposite direction of the response location subjects would eventually choose. That is, if subjects were eventually going to respond long, they were initially pointing more toward the short choice location. Although this effect was not strongly informative of choice, it suggests that the difference in force reflected biases in the initial pointing direction, from which subjects would eventually change their mind.

### Differences in starting location

**Figure 9.**
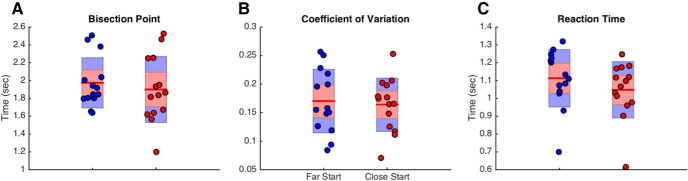
Comparison of behavior for different starting positions. Behavioral results from Experiment 3 (different hold locations) are displayed for the (***A***) bisection point, (***B***) CV, and (***C***) average reaction time across all durations. Red symbols represent individual subjects at the close hold location; blue symbols represent the long hold location. No significant difference between starting location was found for either the bisection point or coefficient of variation, but a significant difference was found for reaction time, with subjects responding sooner when the starting location was closer to the target locations.

As noted above, one explanation for the difference in precision between the hold and free-movement groups is that one group was allowed to be closer to the target locations. As such, the increase in threshold for the free-movement group may simply be the result of them feeling less pressure to make a speeded response, although no difference in RT was found for shorter durations between these groups. To address this possibility, we conducted an additional experiment (*n* = 14) in which subjects performed two blocks of the hold condition, yet with different hold positions, keeping them near or far from the target locations (Fig. 1*C*). If the difference in precision between groups was because of the proximity to the target, then subjects should be more precise in their estimates and have a correspondingly higher threshold when they are held closer to the targets. The results of this experiment demonstrated this was not the case, as no difference between the CV was detected between conditions (Wilcoxon signed ranks test, *Z* = −0.31, *p* = 0.975, 10,000 permutations). No difference between BP values was detected (*p* > 0.05), although a significant difference in RT was found, with subjects responding faster when the hold location was closer to the targets (*Z* = −3.296, *p* < 0.001, Cohen’s *d* = 3.722); we note that no interaction with duration was observed, here, contrary to the difference between free-movement and hold groups in the previous experiments (Fig. 9). These findings suggest that the increase in precision for the free-movement group was not because of the closer proximity these subjects were afforded to the response locations.

### Effect of movement on temporal reproduction

Although the above findings suggest that subjects in the free-movement group are afforded an improvement in temporal precision by moving, which may not be explained by a closer proximity to the target locations, there remain other possibilities to explain this effect. First, during both free-movement and hold experiments, subjects were continuously provided feedback regarding the location of the robotic arm via a cursor projection on the screen. As such, while subjects were timing the auditory stimulus, they were also viewing a visual cue. One possibility, then, is that subjects incorporated this visual stimulus into their estimate of duration. Indeed, previous work has suggested that providing a secondary cue can improve estimates of duration (Ivry and Richardson, 2002). Second, although a difference in CV can be found between free-movement and hold groups, we do not know whether such an improvement would be found within subjects performing both types of task. Last, although the benefit in precision can be found for temporal bisection, it is unclear whether this improvement would also be found in another time estimation task.

**Figure 10.**
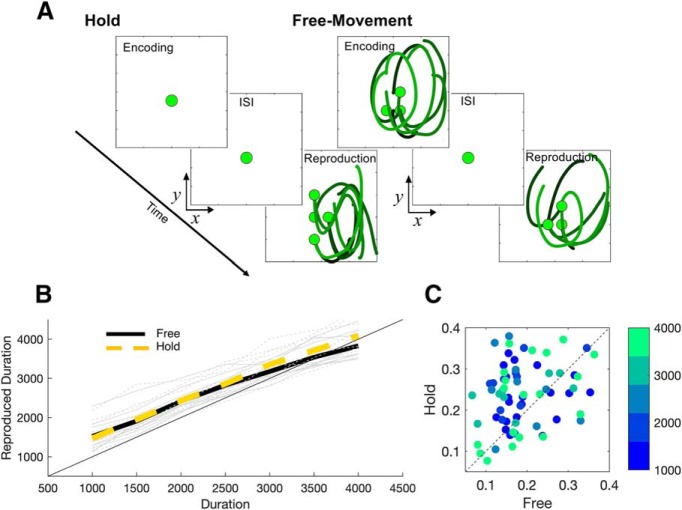
Movement improves temporal reproduction. ***A***, Task schematic for the temporal reproduction task. Subjects (*n* = 10) performed alternating blocks of trials in which they were presented with an auditory stimulus for one of seven possible intervals (1–4 s; encoding), and then required to reproduce that interval (reproduction). In the hold condition, subjects were held in one of six possible starting locations during encoding and the subsequent ISI. In the free-movement condition, subjects were allowed to freely move the arm during the encoding phase, but were forced back to one of the six possible locations when the interval ended. Following the ISI, in either condition, subjects were required to reproduce the interval by moving the robotic arm for the same amount of time as the tone interval they had just heard. No feedback regarding cursor location was presented. Lines within demonstrate seven random trials from a representative subject, with darker lines indicating trials on which shorter intervals were presented. Green circles indicate the starting locations of the respective phases in these trials. ***B***, Temporal reproduction performance. Gray lines indicate individual subject performance; bold lines indicate mean reproduced intervals, with a linear regression fit to these values. Subjects reproduced all intervals well, with a notable central tendency effect between across the stimulus set. A significant difference in the slope of reproduced to presented intervals was found, with free-movement trials exhibiting more central tendency (inset, individual slope values between conditions). ***C***, CV values between conditions. Individual points represent CV values for each of the seven intervals across all 10 subjects. Significantly higher CV values were found across all seven tested durations in the hold condition compared with free-movement, indicating better precision in these trials.

To address the above uncertainties, we had a new set of subjects (*n* = 10) perform a temporal reproduction task (Fig. 10*A*). Temporal reproduction tasks require that subjects encode a presented stimulus duration, and then reproduce that duration so that both times match. In our version of the temporal reproduction task, subjects performed alternating blocks of trials in which they were exposed to an auditory tone lasting for one of seven possible intervals (1–4 s). Crucially, while subjects listened to this interval, they either were allowed to freely move the cursor around (free-movement condition) or were held in one of six possible locations by the robotic arm (hold condition). Following an ISI, the robotic arm moved subjects to a different possible location of the six, and were required to reproduce the interval they had just been exposed to. In both conditions, the method of reproduction was the same: subjects were required to move the cursor for the same length of time as the auditory tone they heard in the encoding phase; the reproduced interval was measured as the difference between when subjects started and stopped moving. Notably, in neither condition were subjects shown a feedback cursor indicating the location of the arm under the projection screen. The change between starting locations in encoding and reproduction phases was done to discourage a strategy of simply repeating the same movement between encoding and reproduction phases.

To analyze these data, we first examined average temporal reproduction performance. Across both conditions, subjects performed the task well, with reproduction performance that closely matched the presented interval. Notably, a central tendency effect was observed (Jazayeri and Shadlen, 2010; Cicchini et al., 2012) in which subjects overestimated the shortest and underestimated the longest interval in the stimulus set, thus regressing their reproductions to the mean of the stimulus set (Fig. 10*B*). This effect was quantified by the slope of a best fitting linear regression for each subject. Notably, a significant difference in slope between conditions was observed, with a shallower slope, indicating greater central tendency, in the free-movement condition (Wilcoxon signed rank test, *Z* = −2.701, *p* = 0.004, Cohen’s *d =* 3.285, 10,000 permutations; Fig. 10*B*). In contrast, we also measured the CV for each of the seven tested intervals. Here, a significant main effect of condition was observed (*F*_(1,9)_ = 9.44, *p* = 0.013, η2p = 0.512), with no interaction across duration (*F*_(6,54_) = 1.468, *p* = 0.207), in which the CV in the hold condition was greater than in the free-movement condition, indicating greater precision in the free-movement group (Fig. 10*C*).

One possible explanation for the improved performance in the free-movement condition is that subjects were simply repeating the same movement during the reproduction phase as in the encoding phase. We discouraged this strategy by changing the starting location between encoding and reproduction phases and by not providing feedback on arm location, yet it is still possible that subjects still attempted to repeat the same movement. To quantify this, we calculated the discrete Fréchet distance between the trajectories in encoding and reproduction phases (Eiter and Manilla, 1994; Alt and Godau, 1995). The Fréchet distance provides an index of the similarity of two polygonal curves; values of zero indicate complete similarity, with higher values indicating greater separation. Formally, this value represents the length of the shortest possible path that can link both curves. Crucially, the Fréchet distance takes into account the ordering of each line; two lines may appear identical, but start and end at opposite locations. We calculated the average discrete Fréchet distance across trials in the free-movement condition for each subject. Here, we found an average value of 0.1596 ± 0.006. A one-sample *t* test revealed these values as significantly different from zero, indicating dissimilarity between trajectories (*t*_(9)_ = 23.631, *p <* 0.001, Cohen’s *d* = 23.6). We additionally investigated whether the Fréchet distance measure changed across the tested intervals; one possibility is that, as the interval becomes longer, a more reliable strategy is to attempt to repeat the movement path during encoding. In other words, although the paths between encoding and reproduction are not identical, they may still be similar. However, we observed no difference in the Fréchet distance across durations (main effect of duration: *F*_(6,54)_ = 1.791, *p* = 0.118), indicating that the similarity of paths between encoding and reproduction phases did not change, further suggesting that subjects were not employing a strategy of reproducing the movement from the encoding phase.

**Figure 11.**
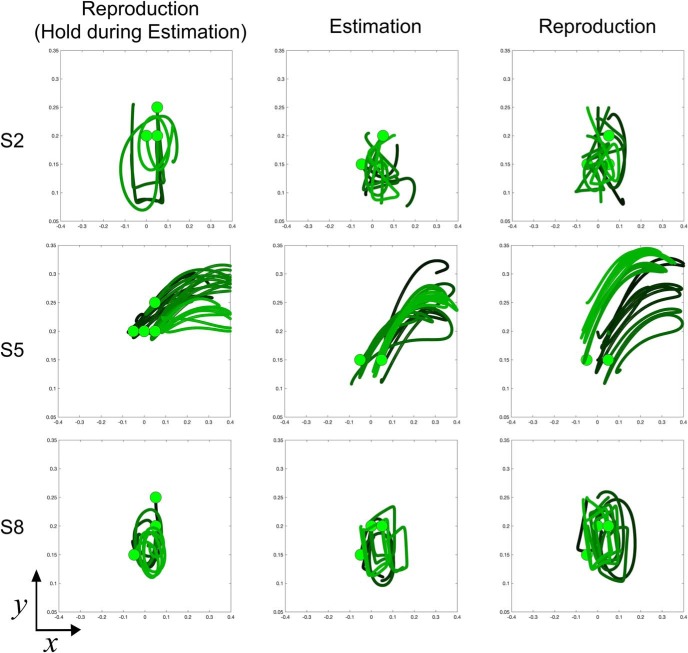
Movement strategies for subjects in the temporal reproduction task. Similar to the temporal bisection task, subjects used a number of different strategies, yet were consistent with the strategy chosen. Three representative subjects are shown with distinct movement strategies across the three moving conditions. Layout is the same as in Figure 10; green circles indicate the starting position. Traces displayed above are from seven trials chosen at random for each subject, with one for each of the seven intervals tested; lighter green colors indicate longer interval trials. Left column, Reproduction movements from trials on which the subject was held in place during the estimation phase. Middle and right columns, Movements made during the estimation and reproduction phases in the free-movement condition. Each subject again adopted a different strategy, such as a random pattern (S2), an arc movement (S5), or a patterned movement consisting of sharp turns (S8).

Observation of the movement trajectories employed by subjects again demonstrated heterogeneity of movement strategies between subjects, yet consistency of strategy within subjects. An additional concern here is that subjects may have employed a “circular” strategy during the encoding phase of the free-movement condition, wherein subjects simply performed a number of circular rotations, and then repeated this number during reproduction. Such a strategy could potentially reduce variability, and would also likely engage an implicit timing mechanism, as circle drawing tasks have been shown to engage (Robertson et al., 1999; Zelaznik et al., 2002; Merchant et al., 2008). However, observation of the movement trajectories (Fig. 11) demonstrated that subjects rarely engaged in circular movements. Instead, we observed subjects engaging either in full “arc” movements, patterned movements consisting of sharp turns or random movements, suggesting a consistent circle-drawing strategy was not used, and further did not convey the precision benefit in this task.

## Discussion

Here, we show that when subjects are allowed to freely move while timing the duration of a concurrent auditory stimulus, they are more precise in their estimates than subjects who are held in place. These findings are likely not because of the closer proximity to the target, motor-based counting strategies, secondary cues, or visual feedback in free-movement subjects; nor are they do to changes-of-mind in hold subjects. Further, we demonstrate that subject choice can be reliably determined by the statistics of movement dynamics before the offset of the interval, but only when subjects are freely allowed to move and not when held in place; further, movement variability increased as timed intervals grew longer, and was notably higher when being held in place. Additionally, we find that force exertion on the arm in subjects held in place can weakly predict eventual choice, but only in the period immediately before trial onset and in the opposite direction of the choice subjects will ultimately choose. Last, we show here that this finding translates to a second type of timing task, in which subjects must reproduce the length of the auditory interval by moving the robotic arm; moving of the arm while encoding the interval again leads to an improvement in precision. These findings suggest that engagement of the motor system confers a perceptual advantage during time estimation and decision-making.

Previous work has begun to demonstrate a strong influence of the motor system on temporal processing. A variety of studies now demonstrate how movement parameters can influence the perceived duration of a stimulus, with longer movements typically associated with longer perceived durations (Hagura et al., 2012; Yokosaka et al., 2015; Yon et al., 2017). Beyond the effect of movement on altering the perception of time, our results here demonstrate an ability to improve it. Previous work by Hagura et al. (2012) has demonstrated that when subjects are preparing to make a movement, temporal fidelity for visual stimuli is improved. Yet, these authors did not analyze or report whether a change in precision also occurs, only a directional shift in the psychophysical function; our findings demonstrate a general improvement in precision when subjects are allowed to move during the timing of an interval. In a separate vein, Tomassini et al. (2015, 2017) have shown an improvement in the perception of visual contrast during movement preparation and execution; however, this improvement varies sinusoidally (∼4 Hz) around the time of movement, suggesting a linkage with visual sampling mechanisms (Busch and VanRullen, 2010). Further work by Yon et al. (2017) demonstrated that concurrent movements while timing a subsecond auditory stimulus, a closer analog to the present task, also shifted the perception of duration, depending on the length, speed, or direction of the movement; however, no overall differences in precision were detected between different movement conditions.

Based on these findings, one explanation is that use of the motor system allowed free-movement subjects to “simulate” the timed interval, taking advantage of the greater temporal fidelity of the motor system. Yet, the timing of auditory stimuli alone is already highly precise, more so than for visual (Wiener et al., 2014) or tactile (Jones et al., 2009) modalities. However, when combined, previous work has demonstrated an improvement over unisensory stimuli alone (Occelli et al., 2011; Manning and Schutz, 2013). Similarly, when tactile information is presented bimodally with visual stimuli, the perception of duration is also improved over unisensory conditions (Ball et al., 2017). Further, experience with audiomotor timing may confer additional advantages; professional drummers are notably less biased and more precise in the timing of both auditory and visual stimuli (Cicchini et al., 2012). Likewise, auditory frequency discrimination performance is impaired when subjects receive stimulation to the somatosensory cortex, but only when they are required to simultaneously attend to touch (Convento et al., 2018). Similarly, recent research has begun to demonstrate that time perception is highly linked to the amplitude and phase of beta (13–30 Hz) oscillations (Arnal et al., 2015; Kulashekhar et al., 2016; Wiener and Kanai, 2016; Morillon and Baillet, 2017; Grabot et al., 2019). That beta oscillations are also highly implicated in movement is likely no coincidence; movement and timing may be intrinsically linked. Moreover, beta oscillations are invoked for the timing of durations even when no timed motor response is required and stimulation at beta frequency (20 Hz) shifts the memory of the perceived durations (Wiener et al., 2018).

If the motor system is intrinsically involved in time perception, then why does the free-movement condition confer an advantage over the hold condition? That is, if timing is already partially instantiated in the motor system, then why should additional movement provide better temporal resolution? In the hold condition, the motor system may be computing parallel yet competing action plans for separate movements to the different target locations, as is the case for other variable reach targets (Cisek and Kalaska, 2010). In the present study, at the start of a trial, the reaching plan to the short target location should be dominant, but as the interval elapses the long target reach plan should gradually start winning, until the internal criterion is passed and subjects decide that the interval is now long; evidence for these competing action plans can be observed in the force direction data from the hold group, which shows a gradual transition, rather than a stepwise shift from short to long. Yet, in the free-movement group, this competition may not necessarily be taking place, as subjects are here able to fluidly explore the 2-dimensional space between targets while sequentially sampling information in favor of one versus the other; that is, in the hold condition, movement preparation precedes initiation, whereas initiation precedes preparation in the free-movement condition (Haith et al., 2016). In this case, noise arising from the competitive process may have less of an influence on the accumulation of evidence (Brunton et al., 2013).

Consistent with the above explanation, we note one previous study that also explored temporal processing and reaching movements, including a temporal bisection task (Méndez et al., 2014). In this study, subjects categorized visual intervals by moving a cursor to one of two different response locations, which could vary around a starting location on a trial-by-trial basis, in a similar setup to the hold condition in the present study. The results of this study found no difference in overall categorization accuracy or precision as a function of response location, yet observed differences in movement time and velocity parameters depending on the interval presented and the response location position. A neuronal network model was subsequently developed that consisted of both accumulator and memory populations, in which the response was governed by network attractor-dynamics, which recapitulated the observed findings. Crucially, the developed network posited that the motor system is both timing and evaluating the tested intervals simultaneously. This suggests that the motor system is likely engaged in both free-movement and hold conditions, as evidenced in the previous study by the results of our logistic regression analysis, but that different strategies are employed between them. In context, the free-movement condition may provide a stronger separation of “correct” and “incorrect” network trajectories.

Despite heterogeneity in the free-movement strategies across subjects, we found that continuous proximity to the target location served as a good predictor of eventual choice. For the middle and most ambiguous interval in the stimulus set, choice location could be determined well before the stimulus offset occurred. Notably, longer movements on these trials more often led subjects to classify the stimulus as long, concordant with previous findings (Yokosaka et al., 2015; Yon et al., 2017). However, even when taking this into account, the divergence between target proximities is striking, with a separation by 1500 ms into the trial that is predictive of choice. Given that subjects do not know that the interval will end at 2000 ms, this suggests that subjects commit to their choice based on proximity to the target at trial offset. As subjects adopted consistent movement strategies throughout the experiment, then the choice of where to respond becomes easier when interval offset occurs. This may in turn translate to a steeper categorization gradient and a smaller coefficient of variation, as observed for free-movement subjects.

For temporal reproduction, we additionally observed an improvement in precision, within the same subjects, when reproducing the duration of a previously encoded auditory stimulus when subjects were allowed to freely move during the encoding phase. When subjects were held in place during encoding of the auditory duration, no benefit of was observed. This effect was observed across all tested durations, and was unlikely to be driven by a simple reproduction of the movement during the encoding phase; the starting location varied between encoding and reproduction phases, encoding and reproduction paths were dissimilar, and subjects received no visual feedback regarding their arm location in either phase. This latter feature also suggests that the benefit observed in the classification experiment was not because of subjects using the visual feedback cursor as a secondary cue to benefit timing performance. Overall, these findings demonstrate that the benefit of movement transfers across different tasks. Crucially, it shows that movement alone does not confer a benefit; as subjects reproduced the interval by moving the robotic arm in both free-movement and hold conditions, then no difference between should be observed between the two if simply moving while reproducing an interval confers a benefit (Rammsayer and Verner, 2015). Instead, subjects must move when actively estimating the interval for there to be a benefit, suggesting that during reproduction subjects are provided with a more precise estimate to work from.

Another consideration from the present data is precisely what stage in the processing hierarchy may be preferentially impacted by movement. That is, could movement specifically be impacting the perception of time, or instead the decision-making capability (Hagura et al., 2017) of the subject? Disentangling the specific role of improvement in a timing task is a non-trivial issue, as multiple factors may simultaneously contribute to the variability of performance (García-Pérez, 2014). However, certain aspects of our study suggest that the source of improvement may lie at the perceptual, rather than decision-making level. First, while the improvement in bisection performance may arise from improved decision-making, this would not necessarily predict an improvement in the temporal reproduction task, where subjects must reproduce an interval from memory. We further note that the crucial manipulation in the reproduction task came during the encoding stage; movement during encoding improved performance at reproduction, which would not be a result of an improved decision fidelity. Second, if the improvement was confined to the decision stage, then one might not expect differences in the variability of movements that scaled with interval duration, as found for free-movement subjects.

As a secondary consideration, one unifying aspect of the various timing tasks employed is the role that sub-vocal counting may have provided subjects, as counting has been demonstrated to also provide a benefit in the precision of timing (Hinton and Rao, 2004); this raises the possibility that subjects were more likely to engage in counting when moving than when being held still. However, in the present study counting was discouraged across all subjects, which has been previously shown to be sufficient to eliminate CV improvements conveyed by counting (Rattat and Droit-Volet, 2012). Further, counting has also been shown to violate the scalar property of timing, in which a constant CV across multiple intervals should be observed. In the temporal reproduction task used here, wherein multiple CVs across duration were collected, we found no change in CV across duration in either condition, suggesting that counting was not the contributor to the change in precision.

In summary, we report here a dissociation in the precision of temporal estimates when subjects are allowed to freely move during the presentation interval compared with being held in place. Our findings cannot be explained by counting strategies, proximity to the target, visual feedback of position, or task specificity, and may depend on an adoption of the motor system to improve the fidelity of categorization and reproduction of perceived time intervals. These results have implications for decision-making and time perception studies, and suggest that the motor system is intrinsically linked to the perception of duration (Merchant and Yarrow, 2016) and that its adoption can aid decision-making (Wolpert and Landy, 2012).

## Synthesis

Reviewing Editor: Satu Palva, University of Helsinki Decisions are customarily a result of the Reviewing Editor and the peer reviewers coming together and discussing their recommendations until a consensus is reached. When revisions are invited, a fact-based synthesis statement explaining their decision and outlining what is needed to prepare a revision will be listed below. The following reviewer(s) agreed to reveal their identity: Hugo Merchant.

This study investigates the effect of reaching movement on the perceptual performance of subjects during an interval categorization task. Subjects moved a robotic manipulandum to one of two locations to categorize a suprasecond interval defined by an auditory stimulus. Two movement conditions were explored: one in which movement during the interval was unrestricted and one in which they were restricted from moving until the stimulus interval had elapsed. The results showed that the categorization precision was larger in the free-moving condition. An additional experiment showed that the reproduction of a single interval was more precise when movements were allowed during interval encoding.

The authors answered properly all the reviewers' comments and now the paper delivers a novel set of findings. The only concern is that the density of arm location during movements in Figure 3 bottom cannot be sufficiently appreciated. A change in color code is needed.

## Author Response

We thank the reviewers for their assessment of our manuscript. We have revised according to comments for this minor revision. Please see below for our responses.

The only concern is that the density of arm location during movements in Figure 3 bottom cannot be sufficiently appreciated. A change in color code is needed.

We have now updated the color code to more clearly show the density of arm location.
